# Plant Bioactive Compounds as an Intrinsic and Sustainable Tool to Enhance the Microbial Safety of Crops

**DOI:** 10.3390/microorganisms9122485

**Published:** 2021-11-30

**Authors:** Andree S. George, Maria T. Brandl

**Affiliations:** Produce Safety and Microbiology Research Unit, United States Department of Agriculture, Agricultural Research Service, Albany, CA 94710, USA; Andree.george@usda.gov

**Keywords:** fruit, vegetable, phytochemical, control, mitigation, antagonism, phenolic, stress, foodborne disease, enteric pathogen

## Abstract

Outbreaks of produce-associated foodborne illness continue to pose a threat to human health worldwide. New approaches are necessary to improve produce safety. Plant innate immunity has potential as a host-based strategy for the deactivation of enteric pathogens. In response to various biotic and abiotic threats, plants mount defense responses that are governed by signaling pathways. Once activated, these result in the release of reactive oxygen and nitrogen species in addition to secondary metabolites that aim at tempering microbial infection and pest attack. These phytochemicals have been investigated as alternatives to chemical sanitization, as many are effective antimicrobial compounds *in vitro*. Their antagonistic activity toward enteric pathogens may also provide an intrinsic hurdle to their viability and multiplication *in planta*. Plants can detect and mount basal defenses against enteric pathogens. Evidence supports the role of plant bioactive compounds in the physiology of *Salmonella enterica*, *Escherichia coli*, and *Listeria monocytogenes* as well as their fitness on plants. Here, we review the current state of knowledge of the effect of phytochemicals on enteric pathogens and their colonization of plants. Further understanding of the interplay between foodborne pathogens and the chemical environment on/in host plants may have lasting impacts on crop management for enhanced microbial safety through translational applications in plant breeding, editing technologies, and defense priming.

## 1. Background and Importance

Outbreaks of produce-associated foodborne illness pose a threat to human health worldwide [1]. *Norovirus, S. enterica,* and *Staphylococcus aureus* are among the most prevalent etiological agents of disease linked to produce [2]. In the European Union, fresh fruits and vegetables are on the highest priority list for new control measures aimed at reducing outbreaks of foodborne disease [3]. Throughout the United States, multistate outbreaks have been on the rise [4,5]. Annual losses from these outbreaks can be in the millions of US dollars, with thousands of lost quality-adjusted life years (QALYs) [6,7]. During the year 2020 in the USA, seven of the ten multistate outbreaks of foodborne illness were confirmed to be associated with produce [8]. Of these outbreaks, all but one were caused by the enteric pathogens *S. enterica,* Shiga-toxin-producing *E. coli,* and *L. monocytogenes*. Of particular concern are outbreaks associated with the consumption of fresh-cut ready-to-eat produce, which presents new opportunities for enteric pathogen colonization due to the inherent presence of compromised tissue.

Despite recurrent outbreaks of foodborne illness associated with fruit and vegetables, the prevalence of enteric pathogens on crops is generally low [9], which may contribute to difficulties in controlling their contamination [10]. Currently, good agricultural and manufacturing practices in conjunction with chemical sanitization during processing are the main control measures available to the produce industry. However, as outbreaks of produce-associated foodborne illness continue to present a threat to human health, it has become clear that these mitigation strategies are insufficient. Furthermore, produce wash water treated with chemical disinfectants has the potential to generate hazardous byproducts [11,12,13,14]. Hence, alternative sustainable approaches to enhancing the microbial safety of fresh fruit and vegetables are direly needed.

In the past two decades, we have achieved a greater understanding of how human pathogens colonize plants. Similar to most bacterial plant colonists, enteric bacterial pathogens tend to aggregate around stomata, trichomes, and cut edges of leafy vegetables [15,16,17,18,19]. The ability of these pathogens to enter plant tissue [20,21,22,23] and form biofilms on plant surfaces [24], thereby resisting efforts aimed at the removal of established colonies, presents important challenges in food safety. Whether bacteria can establish on or within plants depends on various physical, chemical, microbial, and abiotic factors. Some of these factors are influenced by plant genotype, a significant determinant of plant colonization by human pathogens [25,26].

The plant surface presents numerous obstacles to colonization by human pathogens. Physical barriers such as rigid cell walls and waxy cuticles serve as a first defense against foreign invaders. Bacteria must also be equipped to defend against dry conditions and harmful ultraviolet radiation. Furthermore, seasonality and extreme weather events can directly impact surface conditions [27,28], improving or reducing the ability of human pathogens to colonize plant tissue. Once established, bacteria will face competition for nutrients and exposure to inhibitory compounds, including reactive oxygen species (ROS) [29,30]. The phyllosphere native microbiome may itself inhibit the colonization of immigrant cells through competition or the release of antimicrobial compounds [31,32,33]. 

**Figure 1:**
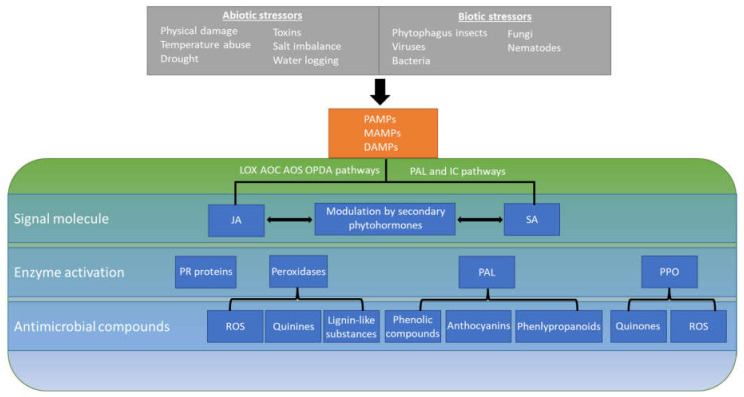
Plant basal defenses and production of antimicrobial compounds. Recognition of pathogen-associated molecular patterns, microbe-associated molecular patterns, and damage-associated molecular patterns (PAMPS, MAMPS, and DAMPS) trigger activation of the jasmonic acid (JA)- and salicylic acid (SA)-mediated defense responses. Through various interconnected pathways, JA and SA, along with secondary phytohormones, lead to an accumulation of peroxidases, phenylalanine ammonia lyase (PAL), polyphenol oxidase (PPO), and the expression of pathogenesis-related (PR) genes, some of which encode defensins. Together, peroxidases, PAL, and PPO are responsible for the generation of compounds important for defending against abiotic and biotic stressors. These pathways and resulting bioactive molecules may be harnessed to control the contamination of plants by enteric pathogens.

Damage to plant tissue in the form of mechanical injury, insect herbivory, and contamination with fungal and bacterial pathogens occurs throughout all phases of crop production. Compromised plant tissue is an important determinant of colonization by enteric pathogens. While *S. enterica, L. monocytogenes,* and *E. coli* are capable of internalizing into plant tissue through various openings [34], their access to damaged tissue has been shown to promote their multiplication and survival [16,35,36,37,38]. Once in contact with plant cells, in the plant apoplast or in injured tissue, enteric pathogens may face a chemical environment at least partly modulated by plant defenses. The plant response to stressors is governed chiefly by jasmonic acid (JA) and salicylic acid (SA) signaling molecules [39,40,41,42]. Activation and suppression of these pathways are further regulated by other phytohormones, which include ethylene, abscisic acid, auxin, gibberellins, cytokinins, and brassinosteroids [43]. The interactions between these pathways and hormones have been recently reviewed [44] and are outside the scope of this review. Once induced, these plant defense molecules lead to signaling cascades that result in physiological changes and the synthesis of defense compounds (summarized in Figure 1). This review presents and discusses (1) the various phytochemicals produced by plants in the context of their defense to biotic and abiotic stresses that may antagonize the viability and multiplication of enteric pathogens in the plant environment, (2) the known mechanisms of antimicrobial activ-ity of some of these compounds, (3) their effect on enteric pathogen behavior and fit-ness on/in plants, and (4) potential applications of phytochemical production in pro-duce safety.

## 2. Basal Immunity against Biotic and Abiotic Stress

### 2.1. Induction of Plant Defenses

The JA signaling pathway is activated in response to abiotic stresses, including physical injury, insect herbivory, and osmotic stress, as well as by elicitors associated with infection by necrotrophic pathogens [39,40,45]. Detection of mechanical wounding or tissue damage begins a signaling cascade with the release of glutamate that culminates in the system-wide release of jasmonates [46,47]. Locally, tissue damage releases JA via the LOX, AOS, AOC, and OPDA signal cascades, resulting in the release of JA and jasmonoyl-L-isoleucine [48,49,50]. Upon release, these jasmonates then coordinate immune responses and the release of defense compounds through binding to the Col1-JAZ coreceptors [49]. In contrast to physical damage, foreign microbes activate the SA and nitrous oxide signaling pathways. Induction of the SA pathway begins upon the recognition of pathogen effectors, termed effector-triggered immunity (ETI), present in the gene-for-gene immune response to recognized plant pathogens along with pathogen-associated molecular patterns, microbe-associated molecular patterns, and damage-associated molecular patterns (PAMPs, MAMPS, and DAMPs, respectively) [51,52,53]. Regardless of the route of induction, activation of these pathways leads to increased expression of pathogenesis-related (PR) genes and their resultant proteins (see Figure 1) [44,54,55,56]. PR genes are involved in the plant defense response and their expression causes an increased level of enzymes necessary for further regulation of the defense response and generation of antimicrobials. While there is a wide array of PR proteins, belonging to over 17 families [57], we will focus here on those relevant to compounds with antibacterial activity.

### 2.2. Generation of Defense Compounds

Together, JA, SA, and the additional modulating phytohormones cause an increase in the enzymes peroxidase, phenylalanine ammonia lyase (PAL), and polyphenol oxidase (PPO). Indeed, relative levels of these enzymes are important markers of plant defense activity. Activation of plant defense pathways and increased levels of these enzymes result in the release of antimicrobial compounds and system-wide physiological changes. These compounds include ROS, quinines, lignin-like substances, phenolic compounds, anthocyanins, and defensins, among others [58]. The increase in production of ROS due to environmental stressors, termed oxidate burst, is one of the earliest plant defense responses and is an effective inhibitor of both fungal and bacterial pathogens [59]. Despite the harmful nature of ROS to plants and other living organisms, they play vital roles in signaling and defense [60]. Superoxide radicals (•O_2_^−^), hydroxyl radicals (•OH), hydrogen peroxide (H_2_O_2_), and singlet oxygen (O_2_) are produced in chloroplasts, mitochondria, peroxisomes, and glyoxysomes [61]. Once activated, receptors of microbial patterns in the plant plasma membrane direct accumulation of ROS in the apoplast [62,63]. 

In addition to ROS, plants produce a variety of phenolic compounds from simple phenolic acids to larger compounds, such as phenylpropanoids, phytoalexins, tannins, flavonoids, and anthocyanins, in response to biotic and abiotic stress [64,65]. These compounds are known to perform a wide range of activities in plants, including defending against human and plant pathogens [66,67,68,69,70,71,72,73]. A recent review has highlighted the role of defensins, another class of PR proteins, and their importance as antimicrobial peptides [74]. Although defensins have largely been recognized as having antibacterial activity against fungal and plant pathogens, synthetic peptides and peptides from *Medicago truncatula* have also shown antimicrobial activity against *E. coli* [75]. Along with the release of defensive compounds, plants will also deposit callose and lignin into cell membranes in an effort to slow the spread of invading pathogens, providing another layer of defense [76,77]. Each of these defense mechanisms has been shown to protect plants against herbivory and plant pathogens, and many bioactive compounds associated with them have the potential to affect colonization by enteric pathogens as well.

## 3. Effect of Plant Compounds on Enteric Pathogens

### 3.1. Plant Extracts

**Figure 2:**
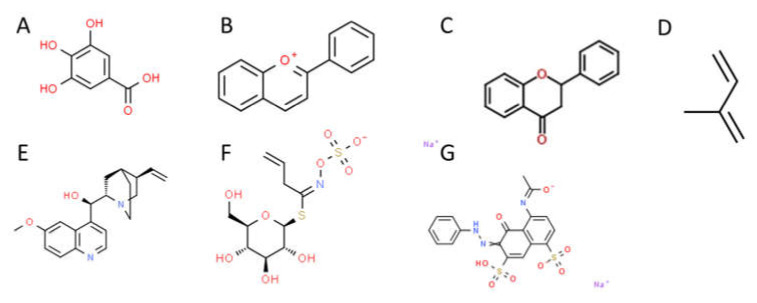
Basic structure of secondary metabolite classes with known antimicrobial properties. (**A**) Tannin (gallic acid). (**B**) Anthocyanin (flavylium). (**C**) Flavonoid. (**D**) Terpenoid (isoprene). (**E**) Alykyloid (quinine). (**F**) Allylglucosinolate. (**G**) Polyphenol (lignin).

Plants produce a variety of secondary metabolites with a wide range of effects. These metabolites include flavonoids, tannins, anthocyanins, terpenoids, alkaloids, glucosinolates, and polyphenolic compounds. Examples of the structure of these different types of compounds can be found in Figure 2. Among their medicinal effects, these compounds are known to have bactericidal and bacteriostatic effects against enteric pathogens under clinical settings in their pure forms [78,79]. There has been increased interest in the efficacy of plant extracts as antimicrobials due to the emergence of antibiotic-resistant pathogens [80]. In this regard, many plant extracts have been proven to be effective antagonists of enteric pathogens. Friedman and co-workers reported on the bactericidal activity of more than 100 essential oils and oil compounds against *Campylobacter jejuni*, *E. coli, L. monocytogenes*, and *S. enterica* *in vitro* [81]. Of those compounds tested, 27 oils and 12 compounds were active against all four pathogens. Carvacrol and cinnamaldehyde, the primary constituents of cinnamon and oregano essential oils, significantly reduced *E. coli* serovar O157:H7 (EcO157) densities on spinach and lettuce when added to wash water [82,83,84]. Kombucha extracts containing mostly catechin and isorhamnetin flavonoids showed antibacterial activity against *Vibrio cholera, Shigella flexneri, S. enterica,* and *E. coli* [85]. Similarly, extracts of cranberry juice and blueberries, both high in phenolic content and anthocyanins, are inhibitory towards *E. coli*, *S. enterica,* and *L. monocytogenes* [86,87,88]. The inhibition of *L. monocytogenes* by plant-derived compounds and essential oils is well-established and has been reviewed recently [89].

### 3.2. Phenolic Compounds

Of great interest in relation to foodborne disease caused by virulent pathogens that produce toxins is that several plant compounds, including polyphenols, can reduce the biological activity or release of toxic molecules, such as Shiga toxins (pathogenic *E. coli*) and enterotoxins (*Staphyloccocus aureus*) [90,91,92]. Research into the efficacy of plant bioactive compounds has, however, provided mixed results. In a survey of 35 polyphenols, Bouarab-Chibane et al. reported that both antibacterial and growth-promoting effects were exhibited by these compounds [93]. Of the 35 compounds tested, 51% either promoted or had negligible effects on the growth of *E. coli*; this number fell to 22% and 9% for *S.enterica* Enteritidis and *L. monocytogenes*, respectively. These results suggest that the interaction between these compounds and pathogenic bacteria, as well as their appropriate dosage, requires further investigation, in particular to ensure that the control of one pathogen does not allow for the amplification of another. 

### 3.3. Reactive Oxygen Species

Reactive oxygen species play an important role in biological systems and must therefore be carefully regulated. In addition to their inhibitory effects, tannins, a subclass of phenolic compounds, generate reactive oxygen species, which can either inhibit *E. coli* growth or protect against subsequent exposure to ROS depending on dosage [94,95]. Whether through exposure from outside sources or produced endogenously, excessive concentrations of ROS are known to damage DNA and interfere with multiple physiological processes in bacteria; this has been well-studied in *E. coli* [96,97]. Hence, the strong antimicrobial potential of ROS has given them consideration as a replacement for synthetic antimicrobials [98]. Owing to their prevalence, enteric pathogens encounter ROS in a wide range of environments. *Salmonella* must survive oxidative burst in macrophages and phagosomes prior to its establishment of the *Salmonella*-containing vacuole [99,100,101]. Besides exposure to toxic oxygen radicals in the animal host, *E. coli* experiences oxidative stress upon predation by free-living protozoa such as *Tetrahymena* [102]. Much evidence exists that enteric pathogens also encounter oxidative stress in the plant environment, as discussed below in Section 5.2. Hence, the generation of ROS along with that of various inhibitory phenolic compounds by plant tissue in response to various stresses presents a framework and rationale for the use of these bioactive molecules to enhance produce safety. 

## 4. Antimicrobial Mode of Action of Plant Polyphenols

Plant polyphenols have multifunctional properties, among which, bactericidal or bacteriostatic effects. The mechanisms of antimicrobial activity of this large class of compounds, which includes flavonoids, phenylpropanoids, and phenolic acids, have been generally poorly studied at the cellular and molecular level. Bouarab-Chibane et al. [93] determined that bacterial species, and not cell wall composition, is the deciding factor in the susceptibility of bacteria to polyphenols. While the mechanism of action of these compounds against enteric pathogens has not been fully explored, it is generally understood that membrane disruption through the accumulation of phenolic compounds on the bacterial surface plays a role [103]. Taguri et al. [104] determined the minimum inhibitory concentration (MIC) of 22 polyphenols in 26 bacterial species and established a relationship between their structure and antibacterial activity. Polyphenols with pyrogallol groups in their structure exhibited strong antibacterial activity, while catechol and resorcinol rings imparted lower activity. Phenols with pyrogallol groups and those with catechols both generate higher H_2_O_2_ at non-acidic pH; thus, other mechanisms may explain differences in their potency against bacterial cells. Although the presence of pyrogallol groups is not a strict requirement for high potency, given that theaflavin and pure catechol, which do not have pyrogallol rings, also showed strong antibacterial activity, these observations provide a basis for in-depth investigations of the interaction of classes of polyphenols with their bacterial target. A large study of 800 plant extracts from different countries identified 12 extracts that inhibited *L. monocytogenes* *in vitro* [105]. Although further characterization of the chemical nature of the bioactive compounds that are responsible for this inhibition is still in progress, SEM revealed that some plant extracts disrupted the cell membrane and/or caused loss of flagella in *L. monocytogenes*, which is in line with the reported mode of action of various phenolic compounds in bacteria. The section below, also summarized in Table 1, describes some of the current information available regarding the effect of major classes of polyphenols on enteric pathogens commonly associated with illness from contaminated crops.

### 4.1. Flavonoids

The flavonoids quercetin, anthocyanins, and other flavonols disrupt the bacterial membrane potential in *E. coli*, and hence membrane integrity, leading to cell leakage [88,106]. This toxicity is thought to result from enzyme inhibition by the oxidized phenolic compounds, including that of membrane-bound proteins, based on the inhibition of plant cell membrane glucan synthase [107]. Quercetin also damaged the cell membrane in *Staphylococcus aureus* [108]. Damage to the lipid bilayer and aggregation in the cell membrane in this pathogen was caused by the catechin derivative epigallocatechin-3-gallate (EGCG) [109], a flavan-3-ol abundant in tea leaves that also binds to enterotoxin B [91]. In contrast, EGCG appeared to produce morphological changes in the cell wall of *E. coli* that are consistent with the release of H_2_O_2_ [109]. Luteolin, which is present in many fruit, vegetables, and herbs, is active against *E. coli*. FT-IR spectroscopy revealed that luteolin effected a cellular increase in fatty acid and nucleic acid, but a decrease in proteins in the bacterial envelope, suggesting that its mode of action involves membrane alteration and protein inhibition [110]. Flavonoids are known to efficiently chelate iron. Thus, it is noteworthy that flavonoids with the pyrogallol group form strong complexes with Fe^2+^ [116], while this pyrogallol group also imparted onto polyphenols the strongest antimicrobial activity in the study by Taguri et al. discussed above [104]. Iron chelation may explain the general antimicrobial activity of flavonoids and variation in the antimicrobial efficacy of members of this family, but evidence to support this common hypothesis is still lacking to date.

### 4.2. Tannins

Fruit such as mango, berries, and especially grapes and pomegranate contain high levels of the hydrolyzable tannins gallotannins and ellagitannins. Although they are effective *in vitro* against common foodborne pathogens that have caused epidemics linked to contaminated produce (*S. enterica*, *E. coli*, and *L. monocytogenes*), their mode of action against these pathogens has not been elucidated. In a large study in which a broad range of foodborne pathogens were tested for their suppression by tannins and 24 different fruit extracts, numerous phenolic compounds were quantified; the total phenol content of the extract, but not the flavonoid-to-total-phenol ratio, was strongly associated with higher antibacterial activity [111]. In particular, mango seed extract and tannic acid containing mostly polygalloyl glucoses had the most potent antibacterial activity. The general antagonistic effect of hydrolyzable tannins was proposed to (1) enzyme inhibition, including that of extracellular enzymes such as glucosyltrans-ferases, (2) reduced microbial growth due to deprivation of substrates, (3) reduced me-tabolism due to the inhibition of oxidative phosphorylation, and (4) iron deprivation [112]. 

### 4.3. Phenylpropanoids

These compounds comprise chlorogenic acids and hydroxycinnamic acids. The latter compounds, namely coumaric, caffeic, and ferulic acids, are among the most widely distributed phenylpropanoids in plants and are produced through the shikimic acid pathway from the aromatic amino acids L-phenylalanine or L-tyrosine [117]. Phenylalanine ammonia-lyase (PAL), which is activated in plants by wounding, among other stressors, further converts L-phenylalanine into trans-cinnamic acid. The latter is then transformed into various phenolic compounds, including coumaric, caffeic, and ferulic acid. The lipophilicity of these weak organic acids allows for their diffusion through bacterial membranes where they subsequently acidify the cytoplasm; it is unclear if cell death is the result of this acidification or of multiple likely physiological disturbances due to the accumulation of the weak acid anion [113]. Their antibacterial activity may vary depending on their degree of hydroxylation and local pH. In *L. monocytogenes,* greater hydroxylation of the cinnamic acid molecule increased its MIC [114], while in another study the degree of hydroxylation of hydroxycinnamic acids only had minor effects on the inhibition of *E. coli*, *Bacillus subtilis*, and *Lactobacillus* spp. [115]. In both studies, lower pH potentiated antibacterial activity, indicating that the environment may be a significant contributor to their efficacy as antimicrobials *in planta*.

### 4.4. Biofilm Formation

Phytochemicals may also prevent, in enteric pathogens, behavior that is critical to their survival on plants and to their resistance to sanitization treatments. As reviewed by Ta and Arnason [118], eugenol and cinnamaldehyde inhibited biofilm formation and inactivated pre-formed biofilms of *L. monocytogenes*, while cinnamaldehyde, resveratrol, quercetin, and epicatechin also reduced biofilms formed by *Cronobacter sakazakii* and *E. coli* O157:H7. In *L. monocytogenes* and *C. sakazakii*, this effect was associated with the downregulation of genes that are critical for biofilm formation. The inhibition of *S. enterica* and *E. coli* motility by the tannin punicalagin and curcumin, respectively, may additionally modulate their behavior on and interactions with, surfaces, possibly including plant surfaces. While gallic acid, methyl gallate, epigallocatechin gallate, and tannic acid all were bactericidal and inhibited swarming motility, the latter three phenolics induced biofilm formation in strains of pathogenic *E. coli* at low concentrations; for tannic acid, this promoting effect may be related to the overexpression of the curli fimbriae genes *csgA*, *csgD*, and *cyaA*. [119]. On the contrary, gallic acid inhibited biofilm formation in all tested pathogenic *E. coli*, irrespective of concentration. This indicated that for certain phytochemicals, concentration may be a major discriminant between a beneficial and antagonistic effect on pathogen behavior. Transcription of the operon *pgaABCD,* which codes for the synthesis and transport of a glucosamine polymer involved in the ability of *E. coli* to produce biofilms *in vitro* and adhere to plants [120], was downregulated by gallic acid at sub-bactericidal levels and may explain its inhibitory effect on biofilm formation [121].

### 4.5. Differential Effects among Pathogens

It is clear from the large body of research on the effect of phytochemicals on foodborne pathogens and other bacterial species of interest to human health that large differences exist in their response to treatment with plant-derived compounds. For example, after 24 h and 60 h of incubation, thymoquinone was most inhibitory against *E. coli*, followed by rutin, epichatechin, and myricetin [68]. By contrast, epichatechin had the lowest minimum inhibitory concentration against *S. enterica*, followed by thymoquinone, rutin, and myricetin. Further investigation of the mode of inhibition and of the specific bacterial targets that interact with given moieties of the phytochemical are critical to a better understanding of these differences or commonalities in function and/or efficacy. Both quercetin and EGCG were reported to affect Gram-positive and -negative bacteria differently. The bacteriostatic effect of quercitin was stronger in *S. aureus* than in *E. coli* and *S. enterica* Typhimurium [108]. Atomic force microscopy of bacterial cells revealed that EGCG caused aggregation in the cell envelope of *S. aureus* followed by cell lysis, whereas temporary grooves and perforations similar to the effect of H_2_O_2_ damage were observed in EcO157. Importantly, EGCG caused similar differences in cell morphology in *Streptococcus mutans* versus *Pseudomonas aeruginosa,* supporting a role for outer cell structure in this causality [109]. On the other hand, the antibacterial activity of 22 polyphenols against 26 bacterial species did not depend on whether the bacterial strains were Gram-positive or -negative; rather, it was species-dependent, with overall weaker activity in members of *Enterobacteriaceae* compared with that in *Aeromonas hydrophila*, *Vibrio parahaemolyticus*, and *V. vulnificus* [104]. Given that the mode of action of phytochemicals frequently results in synergistic antagonism when combined with antibiotics and other antimicrobials [122], knowledge of the chemical and molecular basis for their level of specificity will be essential for developing phytochemical-based approaches to use in produce safety or human health. Furthermore, differences in the efficacy of plant compounds against pathogenic bacteria will be an important consideration in the application of such approaches when ensuring the microbial safety of crops that may be contaminated by multiple pathogens.

## 5. Enteric Pathogen Exposure to Bioactive Compounds *in Planta*

The interaction between human enteric pathogens and plants is a burgeoning area of scientific inquiry, prompted by public health concerns regarding the recurrent outbreaks of foodborne disease associated with the consumption of contaminated fruit and vegetables. Much evidence supports the idea that plants detect *S. enterica* and pathogenic *E. coli* and respond to their presence via basal immunity, which triggers signaling cascades, the expression of defense proteins, oxidative burst, and callose deposition in various plant species [123,124,125,126,127]. Melotto and co-workers recently observed that the phenylpropanoid pathway is induced in lettuce, but not in *A. thaliana*, in response to apoplast colonization by EcO157 and *S*. Typhimurium [128], providing further evidence *in planta* that enteric pathogens are exposed to defense-associated antagonistic phytochemicals in a plant species relevant to produce safety and public health. Thus, in turn, enteric pathogens also respond to chemical cues from the plant environment to facilitate their adaptation and hence survival in this secondary habitat [129]. Phenolic compounds were partly associated with the phyllosphere bacterial community structure of 26 lettuce accessions, especially members of *Enterobacteriaceae* [130], suggesting that phenolics differentially affect plant colonists in shaping the composition of microbial communities. Enteric pathogens are exposed to antagonistic chemicals on the surface and in the apoplast of intact plant tissue due to the presence of plant cell exudates and defense compounds following signaling that microbial colonization is taking place. Furthermore, tissue damage caused by infection and degradation by plant pathogens, herbivory, and mechanical injury during cultivation, harvesting, and processing also result in inhibitory phytochemicals both leaching out of the compromised tissue passively and produced actively in response to physical injury. Molecular and omic approaches in produce safety research, transcriptomics in particular, have contributed to providing evidence that enteric pathogens indeed experience such chemical stresses on plants and in plant tissue. 

### 5.1. Antimicrobial Stress

The *mar* operon controls resistance to multiple antibiotic molecules in *E. coli* and is induced by salicylic acid, a key signaling molecule involved in the plant stress response, by ROS, and by plant-derived phenolics [131,132]. *marA* was upregulated in *E. coli* K12 and in EcO157 strain Sakai on intact lettuce leaves [133,134] as well as in *S. enterica* on tomato shoots and roots [135]. The transcription of this operon and members of its regulon were also activated in EcO157 in lettuce lysate and on cut lettuce leaves, in addition to other genes coding for multidrug efflux pumps, such as *emrD* [136]. *S.* Enteritidis upregulated *emrB* upon treatment with cranberry pomace extract [137], suggesting that this operon likely has a role in sensing antimicrobial stress in multiple enteric pathogens on plants. In cilantro and lettuce leaves macerated by the soft rot pathogen *Dickeya dadantii*, various genes with a potential role as drug efflux pumps and in multidrug resistance were induced in *S.* Typhimurium during its multiplication in degraded leaf tissue [138]. On the other hand, *azoR* (cleavage of aromatic azo compounds), *yhhW* (dioxygenase, degradation of quercetin), and *ygiD* (dioxygenase, catabolism of aromatic compounds) were among the genes whose expression increased the most in *S.* Typhimurium in the latter experimental system, and *azoR* was also upregulated in EcO157 Sakai in lettuce [134]. Transcription of *yhhW* was additionally enhanced during tomato shoot colonization by *S.* Typhimurium [135], indicating that this pathogen may actively catabolize inhibitory phytochemicals on both intact and damaged plant tissue. Linalool, an aromatic terpene present in many plant species, including basil, perforates *E. coli* and *S. enterica* Senftenberg cell membranes and alters motility [139]. Consequently, mutants of *S*. Senftenberg in genes that impart resistance to linalool through chemotaxis (*mcpL*) and lipopolysaccharide production (*rfaG*) had lower survival on basil leaves than their parental strain, demonstrating their role in the fitness of this pathogen in the basil phyllosphere. 

Low-nitrogen stress in tomato plants promoted the accumulation of defense compounds such as rutin and kaempferol-rutinoside, caffeic acid derivatives, and chlorogenic acid in leaves [140,141]. *S. enterica* was not affected in multiplication *in vitro* by the addition of rutin, quercetin, and kaempferol to a culture medium [142]. However, *in planta* studies may be necessary to fully assess the possibility that an enteric pathogen may be inhibited in fruit colonization of tomato plants cultivated under nitrogen limitation, particularly because bacterial physiology is very distinct in culture media and on plants.

Although not explored *in planta* to date, the potentiation of bacterial inhibition by phytochemicals through exposure to additional environmental stresses on plants has been documented. For example, the combined antibacterial activity of UV radiation and caffeic acid, a common plant-derived phenolic, was synergistic *in vitro* against EcO157, *S*. Typhimurium, and *L. monocytogenes* [143]. Furthermore, low pH was a potentiator of the antibacterial activity of cinnamic acid and derivatives in biocide studies [144] as well as in other studies discussed above in Section 4.3. The potentiation of microbial inhibition by the production of phytochemicals in plants may be worthy of investigating in the context of a hurdle approach in produce safety.

### 5.2. Oxidative Stress

Besides aromatic compounds, plant microbial colonists encounter various ROS on plant surfaces, in the apoplast, and in injured tissue. The upregulation of *sodC*, encoding a superoxide dismutase, and of *ycfR* (*bhsA*), encoding a multi-stress resistance protein that is highly inducible in *E. coli* and *S. enterica* under oxidative stress, was observed in EcO157 cells and strain K12 on lettuce leaf surfaces [133]; furthermore, a YcfR mutant of EcO157 strain Sakai had impaired survival on spinach leaves [145]. Increased expression of *sodC* and *ycfR* was further reported in *S*. Typhimurium in tomato shoots and roots [135]. *ycfR* was also upregulated in *S*. Typhimurium in *D. dadantii*-macerated cilantro and lettuce leaves [138] and in EcO157 in lettuce lysate and cut lettuce leaves [136]. Transcriptional analyses in EcO157 in lettuce lysate and cut leaves also revealed that several members of the OxyR regulon, which mediates the response to H_2_O_2_ in *E. coli*, were highly expressed [136]. Additional genes involved in the response to oxidative stress in *S. enterica* and EcO157 were determined to be upregulated by transcriptome analysis in the above model systems and additional ones [134,135,136,138,146].

Several further lines of evidence support the role of plant ROS in the colonization of produce by enteric pathogens. The modulation of H_2_O_2_ released at the site of cut wounds on lettuce showed distinct effects on EcO157 survival [147]. More precisely, heat or Na pyruvate treatment to reduce peroxide concentrations on cut lettuce enhanced colonization by EcO157, while infiltration of the cut leaves with the ROS elicitor harpin reduced its population sizes during storage. Likewise, the multiplication of EcO157 on damaged lettuce leaves was greater when the potent antioxidant ascorbic acid was added to the inoculum than with the non-supplemented inoculum, suggesting that ROS inhibited EcO157 growth in nontreated cut lettuce [148]. On the contrary, ascorbic acid treatment reduced EcO157 growth on spinach leaves in the latter study, perhaps due to secondary effects of the antioxidant on the chemical environment of spinach wounds. Such differences are at play not only at the higher plant taxa level, but also among genotypes of the same plant species. For example, the screening of 11 lettuce genotypes for their epiphytic and apoplastic colonization by EcO157 and *S*. Typhimurium revealed a broad range of bacterial fitness, with lettuce cultivars allowing for high vs. low colonization displaying, respectively, low vs. high oxidative burst and callose deposition [123].

Similarly, from the bacterial side of the interaction, differences in response to plant bioactive compounds exist, as also discussed above in Section 4.5. Among twenty-nine strains of *L. monocytogenes,* there was a positive correlation between their resistance to oxidative stress and their ability to colonize alfalfa, radish, and broccoli sprouts [149]. Overall, these observations point to a central role of the oxidative stress response and ROS detoxification in the physiology of enteric pathogens in the pre- and post-harvest plant habitat.

### 5.3. Nitrosative Stress

The effect of nitrosative stress on enteric pathogens in the plant environment has received relatively little attention despite the known occurrence of NO and RNS produced by plants for essential signaling functions as well as in stress responses. Goudeau et al. [138] showed increased activity of the *S*. Typhimurium NsrR regulon, which is responsible for NO detoxification and nitrosative stress resistance, in cilantro leaves macerated by *D. dadantii*. Micallef and co-workers [135] reported that several genes in the *S.* Typhimurium NsrR regulon had enhanced transcription during the colonization of tomato shoots and roots. In a follow-up study, evidence was obtained that the main nitrosative stress response gene *hmpA*, as well as *yoaG,* were significantly upregulated in *S. enterica* Newport on tomato fruit and leaves compared with similar tissue treated with a NO scavenger [125]. Importantly, greater *S.* Newport population sizes were recovered from NO-scavenged tomato leaves (but not fruit) than from nontreated ones, and the pathogen densities were lower on leaves pre-elicited to produce endogenous NO. The above observations indicate an important role for RNS production in the survival of *S. enterica* on plants. It is, however, unclear if other foodborne pathogens are affected similarly.

## 6. Potential Applications

The treatment of fruit and vegetables with bioactive plant compounds directly on the plant tissue or via addition in the wash water has been reported to decontaminate post-harvest produce with various efficacy, as we mention above in Section 3.1. It is clear also, based on solid lines of evidence discussed herein, that antimicrobial stress imposed onto enteric pathogens passively or actively by the plant *per se* has an important role in their epiphytic and endophytic fitness on/in produce. Thus, the potential for bioactive phytochemicals produced *in situ* as *in planta* tools in produce safety cannot be disregarded and should be thoroughly investigated. The screening of plant varieties has already contributed significantly to improving our knowledge of enteric pathogen—plant interactions to reveal some of the components of defense responses that act as determinants of their colonization ability. Large plant screens, which are a daunting task when human pathogens are part of the experimental system due to biosafety concerns, may nevertheless unveil important new phenotypes and genotypes of great value in crop safety. Newly uncovered traits, some of which may be associated with the production of potent antimicrobial compounds against human pathogens, could be included in plant breeding programs to improve the microbial safety of crops, as has been recently proposed [25]. In tomato, breeding for desirable traits coincidentally selected for genotypes that are more capable of inducing defense pathways in response to tissue damage [150], a promising outcome for the further exploration of these genotypes in the control of enteric pathogens given that they have increased fitness in wounded plant tissue.

Plant technologies can be explored to help plants mount a faster and stronger defense response, including the production of bioactive molecules that would effectively inhibit or kill enteric pathogens. For example, plants can be primed to improve their defensive capability against microbial colonists and abiotic stresses [151]. Priming may be achieved through various means. We have demonstrated that priming through induced systemic resistance and systemic acquired resistance lower the growth and survival of *S. enterica* in the lettuce and basil leaf apoplast [152]. Treatment with beneficial bacteria that produce quorum sensing molecules can also serve the function of priming plant defenses. *A. thaliana* plants grown in soil inoculated with *Ensifer meliloti* producing oxo-C14-homoserinelactone were more resistant to colonization by *S. enterica* [153]. Plants that are exogenously provided ethylene, another form of priming, inhibit colonization by *S. enterica* compared with plants that are insensitive to ethylene [154,155]. Gene editing was also shown to be an effective method for enhancing plant defense responses to invading bacteria [156]. The new gene editing tool CRISPR/Cas is being explored in plant–microbe interactions to improve resistance to plant pathogens [157]. Mutants of *A. thaliana* with increased PAMP-triggered immunity were constructed with CRISPR/Cas [158]. Hence, gene editing technologies that suppress enteric pathogens on plants via the enhanced or altered production of defense-based phytochemicals may offer novel approaches to improve crop safety.

## 7. Conclusions and Perspectives

While plant bioactive compounds have been of major interest for their nutritional and medicinal properties, investigation into their efficacy in reducing the microbial contamination of plants that are recurrently associated with outbreaks of foodborne disease has been thus far very limited. Most studies have focused on the inactivation of enteric pathogens by applying phytochemicals into bacterial cultures or onto plant surfaces to assess their potential as sanitization agents. From the knowledge acquired to date about the interaction of enteric pathogens with plants, it is clear that these human pathogens are sensed by plant basal immunity systems and that they reciprocally respond physiologically to plant defenses. However, research must be pursued to gain a deeper understanding of the specific plant pathways and molecules involved in the potentially deleterious effect of immunity on enteric pathogens. This field of research will benefit from a more extensive use of plant genetic and molecular approaches to provide solid evidence for pathways that antagonize enteric pathogens on plants and the mechanisms at the root of enteric pathogen recognition. Likewise, chemical and molecular approaches will provide much needed insight into the antibacterial function of phytochemicals. Once both sides of this interaction are better understood, it may be feasible to manipulate plant pathways such that their resulting products are more potent antimicrobials and more specifically and locally effective against enteric pathogens.

While plant breeding, gene editing, and the priming of plant defenses are promising avenues for reducing human pathogens on plants, the complexity of these systems and the variety of plant–microbe interactions will require a multi-faceted approach. Given that there is zero tolerance for the presence of foodborne pathogens in produce, it is imperative that any single mitigation that cannot guarantee the inactivation of every enteric pathogen cell be part of hurdles that will decrease the probability of a pathogen be-coming established on the plant tissue. Hence, the production of phytochemicals as intrinsic deterrents of enteric pathogen survival and colonization of edible crops must be valued as a strategy in a spectrum of hurdle technologies that aim at enhancing the microbial safety of fresh fruit and vegetables. 

## Figures and Tables

**Table 1:** Mode of action of phytochemicals with known inhibitory activity against enteric pathogens that caused foodborne illness from contaminated produce.

Class	Bioactive Compound	Mode of Action	Antimicrobial Activity Against	Reference
**Flavonoids**	Quercetin	Disruption of cell membrane integrity leading to cell leakage. Enzyme inhibition	*E. coli* *S. aureus*	[106,107,108]
	Epigallocatechin-3-gallate	Damage to lipid bilayer	*E. coli* *S. aureus*	[109]
	Luteolin	Membrane alteration and protein inhibition	*E. coli*	[110]
**Tannins**	Gallotannins Ellagitannins	Enzyme inhibition, substrate deprivation, metabolism reduction, and Fe deprivation	*S. enterica* *E. coli* *L. monocytogenes*	[111,112]
**Phenylpropanoids**	Coumaric acid Caffeic acid Ferulic acid	Acidification of cytoplasm, physiological disturbances due to accumulation of weak acid anion	*L. monocytogenes* *E. coli* *B. Subtilis* *Lactobacillus spp.*	[113,114,115]

## Data Availability

Not applicable.

## References

[1] Jahan S. (2012). Epidemiology of foodborne illness. Sci. Health Social Aspects Food Ind..

[2] Li M., Baker C.A., Danyluk M.D., Belanger P., Boelaert F., Cressey P., Gheorghe M., Polkinghorne B., Toyofuku H., Havelaar A.H. (2018). Identification of biological hazards in produce consumed in industrialized countries: A review. J. Food Prot..

[3] Hackl E., Hölzl C., Konlechner C., Sessitsch A. (2013). Food of plant origin: Production methods and microbiological hazards linked to food-borne disease. Reference: CFT/EFSA/BIOHAZ/2012/01 Lot 1 (Food of plant origin with high water content such as fruits, vegetables, juices and herbs). EFSA Support. Pub..

[4] Gould L.H., Walsh K.A., Vieira A.R., Herman K., Williams I.T., Hall A.J., Cole D. (2013). Surveillance for foodborne disease outbreaks - United States, 1998–2008. MMWR Surveill. Summ..

[5] Carstens C.K., Salazar J.K., Darkoh C. (2019). Multistate outbreaks of foodborne illness in the United States associated with fresh produce from 2010 to 2017. Front. Microbiol..

[6] Morris J.G., Hoffmann S., Batz B. (2011). Ranking the Risks: The 10 Pathogen-Food Combinations with the Greatest Burden on Public Health. http://hdl.handle.net/10244/1022.

[7] Hussain M.A., Dawson C.O. (2013). Economic impact of food safety outbreaks on food businesses. Foods.

[8] Centers for Disease Control and Prevention List of Selected Multistate Foodborne Outbreak Investigations. https://www.cdc.gov/foodsafety/outbreaks/multistate-outbreaks/outbreaks-list.html.

[9] De Oliveira Elias S., Noronha T.B., Tondo E.C. (2019). *Salmonella spp*. and *Escherichia coli* O157:H7 prevalence and levels on lettuce: A systematic review and meta-analysis. Food Microbiol..

[10] Murray K., Wu F., Shi J., Jun Xue S., Warriner K. (2017). Challenges in the microbiological food safety of fresh produce: Limitations of post-harvest washing and the need for alternative interventions. Food Qual. Saf..

[11] Lee W.-N., Huang C.-H. (2019). Formation of disinfection byproducts in wash water and lettuce by washing with sodium hypochlorite and peracetic acid sanitizers. Food Chem.: X.

[12] Gadelha J., Allende A., Galvez F.L., Fernandez P.S., Gil M.I., Egea J.A., Gadelha S.J.R. (2019). Chemical risks associated with ready-to-eat vegetables: Quantitative analysis to estimate formation and/or accumulation of disinfection byproducts during washing. EFSA J..

[13] Fan X., Sokorai K.J. (2015). Formation of trichloromethane in chlorinated water and fresh-cut produce and as a result of reaction with citric acid. Postharvest Biol. Technol..

[14] Zang T., Lee W.-N., Luo Y., Huang C.-H. (2021). Flume and single-pass washing systems for fresh-cut produce processing: Disinfection by-products evaluation. Food Control.

[15] Brandl M.T., Mandrell R.E. (2002). Fitness of *Salmonella enterica* serovar Thompson in the cilantro phyllosphere. Appl. Environ. Microbiol..

[16] Brandl M.T. (2008). Plant lesions promote the rapid multiplication of *Escherichia coli* O157:H7 on postharvest lettuce. Appl. Environ. Microbiol..

[17] Barak J.D., Kramer L.C., Hao L.-Y. (2011). Colonization of tomato plants by *Salmonella enterica* is cultivar dependent, and type 1 trichomes are preferred colonization sites. Appl. Environ. Microbiol..

[18] Melotto M., Underwood W., Koczan J., Nomura K., He S.Y. (2006). Plant stomata function in innate immunity against bacterial invasion. Cell.

[19] Seo K., Frank J. (1999). Attachment of *Escherichia coli* O157: H7 to lettuce leaf surface and bacterial viability in response to chlorine treatment as demonstrated by using confocal scanning laser microscopy. J. Food Prot..

[20] Gu G., Hu J., Cevallos-Cevallos J.M., Richardson S.M., Bartz J.A., van Bruggen A.H. (2011). Internal colonization of *Salmonella enterica* serovar Typhimurium in tomato plants. PLoS ONE.

[21] Wright K.M., Crozier L., Marshall J., Merget B., Holmes A., Holden N.J. (2017). Differences in internalization and growth of *Escherichia coli* O157: H7 within the apoplast of edible plants, spinach and lettuce, compared with the model species *Nicotiana benthamiana*. Microb. Biotechnol..

[22] Turner A.N., Friedrich L.M., Danyluk M.D. (2016). Influence of temperature differential between tomatoes and postharvest water on *Salmonella* internalization. J. Food Prot..

[23] Hora R., Warriner K., Shelp B.J., Griffiths M.W. (2005). Internalization of *Escherichia coli* O157:H7 following biological and mechanical disruption of growing spinach plants. J. Food Prot..

[24] Yaron S., Romling U. (2014). Biofilm formation by enteric pathogens and its role in plant colonization and persistence. Microb. Biotechnol..

[25] Melotto M., Brandl M.T., Jacob C., Jay-Russell M.T., Micallef S.A., Warburton M.L., Van Deynze A. (2020). Breeding crops for enhanced food safety. Front. Plant Sci..

[26] Jechalke S., Schierstaedt J., Becker M., Flemer B., Grosch R., Smalla K., Schikora A. (2019). *Salmonella* Establishment in agricultural soil and colonization of crop plants depend on soil type and plant species. Front. Microbiol..

[27] Ge C., Lee C., Lee J. (2012). The impact of extreme weather events on *Salmonella* internalization in lettuce and green onion. Food Res. Int..

[28] Marvasi M., Hochmuth G.J., Giurcanu M.C., George A.S., Noel J.T., Bartz J., Teplitski M. (2013). Factors that affect proliferation of *Salmonella* in tomatoes post-harvest: The roles of seasonal effects, irrigation regime, crop and pathogen genotype. PLoS ONE.

[29] Wilson M., Lindow S.E. (1994). Coexistence among epiphytic bacterial populations mediated through nutritional resource partitioning. Appl. Environ. Microbiol..

[30] L’haridon F., Besson-Bard A., Binda M., Serrano M., Abou-Mansour E., Balet F., Schoonbeek H.-J., Hess S., Mir R., Léon J. (2011). A permeable cuticle is associated with the release of reactive oxygen species and induction of innate immunity. PLoS Pathog..

[31] Ongena M., Jacques P. (2008). *Bacillus* lipopeptides: Versatile weapons for plant disease biocontrol. Trends Microbiol..

[32] Liu H., Brettell L.E., Qiu Z., Singh B.K. (2020). Microbiome-mediated stress resistance in plants. Trends Plant Sci..

[33] Klerks M.M., Franz E., van Gent-Pelzer M., Zijlstra C., van Bruggen A.H. (2007). Differential interaction of *Salmonella enterica* serovars with lettuce cultivars and plant-microbe factors influencing the colonization efficiency. ISME J..

[34] Standing T.-A., du Plessis E., Duvenage S., Korsten L. (2013). Internalisation potential of *Escherichia coli* O157: H7, *Listeria monocytogenes*, *Salmonella enterica* subsp. enterica serovar Typhimurium and *Staphylococcus aureus* in lettuce seedlings and mature plants. J. Water Health.

[35] Wells J., Butterfield J. (1997). *Salmonella* contamination associated with bacterial soft rot of fresh fruits and vegetables in the marketplace. Plant Dis..

[36] Harapas D., Premier R., Tomkins B., Franz P., Ajlouni S. (2010). Persistence of *Escherichia coli* on injured vegetable plants. Int. J. Food Microbiol..

[37] Koukkidis G., Haigh R., Allcock N., Jordan S., Freestone P. (2017). Salad leaf juices enhance *Salmonella* growth, colonization of fresh produce, and virulence. Appl. Environ. Microbiol..

[38] Smith A., Moorhouse E., Monaghan J., Taylor C., Singleton I. (2018). Sources and survival of *Listeria monocytogenes* on fresh, leafy produce. J. Appl. Microbiol..

[39] Hickman R., Van Verk M.C., Van Dijken A.J.H., Mendes M.P., Vroegop-Vos I.A., Caarls L., Steenbergen M., Van der Nagel I., Wesselink G.J., Jironkin A. (2017). Architecture and dynamics of the jasmonic acid gene regulatory network. Plant Cell.

[40] Ruan J., Zhou Y., Zhou M., Yan J., Khurshid M., Weng W., Cheng J., Zhang K. (2019). Jasmonic acid signaling pathway in plants. Int. J. Mol. Sci..

[41] An C., Mou Z. (2011). Salicylic acid and its function in plant immunity. J. Integr. Plant Biol..

[42] Pieterse C.M., Van der Does D., Zamioudis C., Leon-Reyes A., Van Wees S.C. (2012). Hormonal modulation of plant immunity. Annu. Rev. Cell. Dev. Biol..

[43] Shigenaga A.M., Argueso C.T. (2016). No hormone to rule them all: Interactions of plant hormones during the responses of plants to pathogens. Proc. Semin. Cell Dev. Biol..

[44] Berens M.L., Berry H.M., Mine A., Argueso C.T., Tsuda K. (2017). Evolution of hormone signaling networks in plant defense. Annu. Rev. Phytopathol..

[45] Howe G.A., Jander G. (2008). Plant immunity to insect herbivores. Annu. Rev. Plant Biol..

[46] Mousavi S.A., Chauvin A., Pascaud F., Kellenberger S., Farmer E.E. (2013). GLUTAMATE RECEPTOR-LIKE genes mediate leaf-to-leaf wound signalling. Nature.

[47] Toyota M., Spencer D., Sawai-Toyota S., Jiaqi W., Zhang T., Koo A.J., Howe G.A., Gilroy S. (2018). Glutamate triggers long-distance, calcium-based plant defense signaling. Science.

[48] Herde M., Koo A.J., Howe G.A. (2013). Elicitation of jasmonate-mediated defense responses by mechanical wounding and insect herbivory. Jasmonate Signaling.

[49] Howe G.A., Major I.T., Koo A.J. (2018). Modularity in jasmonate signaling for multistress resilience. Annu. Rev. Plant Biol..

[50] Turner J.G., Ellis C., Devoto A. (2002). The jasmonate signal pathway. Plant Cell.

[51] DebRoy S., Thilmony R., Kwack Y.B., Nomura K., He S.Y. (2004). A family of conserved bacterial effectors inhibits salicylic acid-mediated basal immunity and promotes disease necrosis in plants. Proc. Natl. Acad. Sci. USA.

[52] Van Wees S.C., Van der Ent S., Pieterse C.M. (2008). Plant immune responses triggered by beneficial microbes. Curr. Opin. Plant Biol..

[53] Benedetti M., Pontiggia D., Raggi S., Cheng Z., Scaloni F., Ferrari S., Ausubel F.M., Cervone F., De Lorenzo G. (2015). Plant immunity triggered by engineered *in vivo* release of oligogalacturonides, damage-associated molecular patterns. Proc. Natl. Acad. Sci. USA.

[54] Chen Z., Malamy J., Henning J., Conrath U., Sanchez-Casas P., Silva H., Ricigliano J., Klessig D.K. (1995). Induction, modification, and transduction of the salicylic acid signal in plant defense responses. Proc. Natl. Acad. Sci. USA.

[55] Klessig D.F., Durner J., Noad R., Navarre D.A., Wendehenne D., Kumar D., Zhou J.M., Shah J., Zhang S., Kachroo P. (2000). Nitric oxide and salicylic acid signaling in plant defense. Proc. Natl. Acad. Sci. USA.

[56] War A.R., Paulraj M.G., War M.Y., Ignacimuthu S. (2011). Role of salicylic acid in induction of plant defense system in chickpea (Cicer arietinum L.). Plant Signal. Behav..

[57] Prasannath K. (2017). Plant defense-related enzymes against pathogens: A review. J. Agric. Sci..

[58] Van Loon L.C., Van Strien E. (1999). The families of pathogenesis-related proteins, their activities, and comparative analysis of PR-1 type proteins. Physiol. Mol. Plant Pathol..

[59] Shetty N.P., Jørgensen H.J.L., Jensen J.D., Collinge D.B., Shetty H.S. (2008). Roles of reactive oxygen species in interactions between plants and pathogens. Eur. J. Plant Pathol..

[60] Sharma P., Jha A.B., Dubey R.S., Pessarakli M. (2012). Reactive oxygen species, oxidative damage, and antioxidative defense mechanism in plants under stressful conditions. J. Bot..

[61] Singh R., Singh S., Parihar P., Mishra R.K., Tripathi D.K., Singh V.P., Chauhan D.K., Prasad S.M. (2016). Reactive oxygen species (ROS): Beneficial companions of plants’ developmental processes. Front. Plant Sci..

[62] Qi J., Wang J., Gong Z., Zhou J.M. (2017). Apoplastic ROS signaling in plant immunity. Curr. Opin. Plant Biol..

[63] Boller T., Felix G. (2009). A renaissance of elicitors: Perception of microbe-associated molecular patterns and danger signals by pattern-recognition receptors. Annu. Rev. Plant Biol..

[64] Bhattacharya A., Sood P., Citovsky V. (2010). The roles of plant phenolics in defence and communication during *Agrobacterium* and *Rhizobium* infection. Mol. Plant Pathol..

[65] Rehman F., Khan F., Badruddin S. (2012). Role of phenolics in plant defense against insect herbivory. Chemistry of Phytopotentials: Health, Energy and Environmental Perspectives.

[66] Ravn H., Andary C., Kovács G., Mølgaard P. (1989). Caffeic acid esters as *in vitro* inhibitors of plant pathogenic bacteria and fungi. Biochem. Syst. Ecol..

[67] Rauha J.P., Remes S., Heinonen M., Hopia A., Kahkonen M., Kujala T., Pihlaja K., Vuorela H., Vuorela P. (2000). Antimicrobial effects of Finnish plant extracts containing flavonoids and other phenolic compounds. Int. J. Food Microbiol..

[68] Cetin-Karaca H., Newman M.C. (2015). Antimicrobial efficacy of plant phenolic compounds against *Salmonella* and *Escherichia coli*. Food Biosci..

[69] Takó M., Kerekes E.B., Zambrano C., Kotogán A., Papp T., Krisch J., Vágvölgyi C. (2020). Plant phenolics and phenolic-enriched extracts as antimicrobial agents against food-contaminating microorganisms. Antioxidants.

[70] Proestos C., Boziaris I., Nychas G.-J., Komaitis M. (2006). Analysis of flavonoids and phenolic acids in Greek aromatic plants: Investigation of their antioxidant capacity and antimicrobial activity. Food Chem..

[71] Puupponen-Pimia R., Nohynek L., Meier C., Kahkonen M., Heinonen M., Hopia A., Oksman-Caldentey K.M. (2001). Antimicrobial properties of phenolic compounds from berries. J. Appl. Microbiol..

[72] Cho M.H., Lee S.W. (2015). Phenolic phytoalexins in rice: Biological functions and biosynthesis. Int. J. Mol. Sci..

[73] Kurosaki F., Nishi A. (1983). Isolation and antimicrobial activity of the phytoalexin 6-methoxymellein from cultured carrot cells. Phytochemistry.

[74] Sathoff A.E., Samac D.A. (2019). Antibacterial activity of plant defensins. Mol. Plant Microbe Interact..

[75] Sathoff A.E., Velivelli S., Shah D.M., Samac D.A. (2019). Plant defensin peptides have antifungal and antibacterial activity against human and plant pathogens. Phytopathology.

[76] Luna E., Pastor V., Robert J., Flors V., Mauch-Mani B., Ton J. (2011). Callose deposition: A multifaceted plant defense respons. Mol. Plant Microbe Interact..

[77] Wang Y., Li X., Fan B., Zhu C., Chen Z. (2021). Regulation and function of defense-related callose deposition in plants. Int. J. Mol. Sci..

[78] Omojate Godstime C., Enwa F.O., Jewo A.O., Eze C.O. (2014). Mechanisms of antimicrobial actions of phytochemicals against enteric pathogens–a review. J. Pharm. Chem. Biol. Sci..

[79] Enwa F., Omojate C., Adonu C. (2013). A review on the phytochemical profile and the antibacterial susceptibility pattern of some clinical isolates to the ethanolic leaves extract of *Moringa oleifera* Lam (*Moringaceae*). Int. J. Adv. Res..

[80] Friedman M. (2015). Antibiotic-resistant bacteria: Prevalence in food and inactivation by food-compatible compounds and plant extracts. J. Agric. Food Chem..

[81] Friedman M., Henika P.R., Mandrell R.E. (2002). Bactericidal activities of plant essential oils and some of their isolated constituents against *Campylobacter jejuni*, *Escherichia coli*, *Listeria monocytogenes*, and *Salmonella enterica*. J. Food Prot..

[82] Yossa N., Patel J., Millner P., Ravishankar S., Lo Y.M. (2013). Antimicrobial activity of plant essential oils against *Escherichia coli* O157:H7 and *Salmonella* on lettuce. Foodborne Pathog. Dis..

[83] Yossa N., Patel J., Millner P., Lo Y.M. (2012). Essential oils reduce *Escherichia coli* O157:H7 and *Salmonella* on spinach leaves. J. Food Prot..

[84] Denton J.J., Ravishankar S., Friedman M., Jaroni D. (2015). Efficacy of plant-derived compounds against *Escherichia coli* O157:H7 during flume-washing and storage of organic leafy greens. J. Food Process. Preserv..

[85] Bhattacharya D., Bhattacharya S., Patra M.M., Chakravorty S., Sarkar S., Chakraborty W., Koley H., Gachhui R. (2016). Antibacterial activity of polyphenolic fraction of kombucha against enteric bacterial pathogens. Curr. Microbiol..

[86] Côté J., Caillet S., Doyon G., Dussault D., Sylvain J.-F., Lacroix M. (2011). Antimicrobial effect of cranberry juice and extracts. Food Control.

[87] Shen X., Sun X., Xie Q., Liu H., Zhao Y., Pan Y., Hwang C.-A., Wu V.C. (2014). Antimicrobial effect of blueberry (*Vaccinium corymbosum* L.) extracts against the growth of *Listeria monocytogenes* and *Salmonella* Enteritidis. Food Control.

[88] Lacombe A., Wu V.C., Tyler S., Edwards K. (2010). Antimicrobial action of the American cranberry constituents; phenolics, anthocyanins, and organic acids, against *Escherichia coli* O157:H7. Int. J. Food Microbiol..

[89] Kawacka I., Olejnik-Schmidt A., Schmidt M., Sip A. (2021). Natural plant-derived chemical compounds as *Listeria monocytogenes* inhibitors *in vitro* and in food 
model systems. Pathogens.

[90] Friedman M., Rasooly R. (2013). Review of the inhibition of biological activities of food-related selected toxins by natural compounds. Toxins.

[91] Hisano M., Yamaguchi K., Inoue Y., Ikeda Y., Iijima M., Adachi M., Shimamura T. (2003). Inhibitory effect of catechin against the superantigen staphylococcal enterotoxin B (SEB). Arch. Dermatol. Res..

[92] Quiñones B., Massey S., Friedman M., Swimley M.S., Teter K. (2009). Novel cell-based method to detect Shiga toxin 2 from *Escherichia coli* O157: H7 and inhibitors of toxin activity. Appl. Environ. Microbiol..

[93] Bouarab-Chibane L., Forquet V., Lanteri P., Clement Y., Leonard-Akkari L., Oulahal N., Degraeve P., Bordes C. (2019). Antibacterial properties of polyphenols: Characterization and QSAR (Quantitative Structure-Activity Relationship) models. Front. Microbiol..

[94] Min E.R., Pinchak W.E., Anderson R.C., Callaway T.R. (2007). Effect of tannins on the *in vitro* growth of *Escherichia coli* O157:H7 and *in vivo* growth of generic *Escherichia coli* excreted from steers. J. Food Prot..

[95] Smirnova G.V., Samoylova Z.Y., Muzyka N.G., Oktyabrsky O.N. (2009). Influence of polyphenols on *Escherichia coli* resistance to oxidative stress. Free Radic. Biol. Med..

[96] Imlay J.A., Linn S. (1988). DNA damage and oxygen radical toxicity. Science.

[97] Storz G., Imlay J.A. (1999). Oxidative stress. Curr. Opin. Microbiol..

[98] Dryden M. (2018). Reactive oxygen species: A novel antimicrobial. Int. J. Antimicrob. Agents.

[99] Fang F.C. (2011). Antimicrobial actions of reactive oxygen species. mBio.

[100] Van der Heijden J., Bosman E.S., Reynolds L.A., Finlay B.B. (2015). Direct measurement of oxidative and nitrosative stress dynamics in *Salmonella* inside macrophages. Proc. Natl. Acad. Sci. USA.

[101] Hebrard M., Viala J.P., Meresse S., Barras F., Aussel L. (2009). Redundant hydrogen peroxide scavengers contribute to *Salmonella* virulence and oxidative stress resistance. J. Bacteriol..

[102] George A.S., Rehfuss M.Y.M., Parker C.T., Brandl M.T. (2020). The transcriptome of *Escherichia coli* O157: H7 reveals a role for oxidative stress resistance in its survival from predation by *Tetrahymena*. FEMS Microbiol. Ecol..

[103] Negi P.S. (2012). Plant extracts for the control of bacterial growth: Efficacy, stability and safety issues for food application. Int. J. Food Microbiol..

[104] Taguri T., Tanaka T., Kouno I. (2006). Antibacterial spectrum of plant polyphenols and extracts depending upon hydroxyphenyl structure. Biol. Pharm. Bull..

[105] Ceruso M., Clement J.A., Todd M.J., Zhang F., Huang Z., Anastasio A., Pepe T., Liu Y. (2020). The inhibitory effect of plant extracts on growth of the foodborne pathogen, *Listeria monocytogenes*. Antibiotics.

[106] Qu S., Dai C., Shen Z., Tang Q., Wang H., Zhai B., Zhao L., Hao Z. (2019). Mechanism of synergy between tetracycline and quercetin against antibiotic resistant *Escherichia coli*. Front. Microbiol..

[107] Mason T., Wasserman B. (1987). Inactivation of red beet beta-glucan synthase by native and oxidized phenolic compounds. Phytochemistry.

[108] Wang S., Yao J., Zhou B., Yang J., Chaudry M.T., Wang M., Xiao F., Li Y., Yin W. (2017). Bacteriostatic effect of quercetin as an antibiotic alternative *in vivo* and its antibacterial mechanism *in vitro*. J. Food Prot..

[109] Cui Y., Oh Y., Lim J., Youn M., Lee I., Pak H., Park W., Jo W., Park S. (2012). AFM study of the differential inhibitory effects of the green tea polyphenol (−)-epigallocatechin-3-gallate (EGCG) against Gram-positive and Gram-negative bacteria. Food Microbiol..

[110] Eumkeb G., Siriwong S., Thumanu K. (2012). Synergistic activity of luteolin and amoxicillin combination against amoxicillin-resistant *Escherichia coli* and mode of action. J. Photochem. Photobiol. B Biology.

[111] Widsten P., Cruz C.D., Fletcher G.C., Pajak M.A., McGhie T.K. (2014). Tannins and extracts of fruit byproducts: Antibacterial activity against foodborne bacteria and antioxidant capacity. J. Agric. Food Chem..

[112] Scalbert A. (1991). Antimicrobial properties of tannins. Phytochemistry.

[113] Hirshfield I.N., Terzulli S., O’Byrne C. (2003). Weak organic acids: A panoply of effects on bacteria. Sci. Prog..

[114] Wen A., Delaquis P., Stanich K., Toivonen P. (2003). Antilisterial activity of selected phenolic acids. Food Microbiol..

[115] Sánchez-Maldonado A., Schieber A., Gänzle M. (2011). Structure–function relationships of the antibacterial activity of phenolic acids and their metabolism by lactic acid bacteria. J. Appl. Microbiol..

[116] Porfírio D.A., de Queiroz Ferreira R., Malagutti A.R., Valle E.M.A. (2014). Electrochemical study of the increased antioxidant capacity of flavonoids through complexation with iron (II) ions. Electrochim. Acta.

[117] Rice-Evans C.A., Miller N.J., Paganga G. (1996). Structure-antioxidant activity relationships of flavonoids and phenolic acids. Free Radical Biol. Med..

[118] Ta C.A.K., Arnason J.T. (2016). Mini review of phytochemicals and plant taxa with activity as microbial biofilm and quorum sensing inhibitors. Molecules.

[119] Dávila-Aviña J., Gil-Solís C., Merino-Mascorro J., García S., Heredia N. (2020). Phenolics with bactericidal activity alter motility and biofilm formation in enterotoxigenic, enteropathogenic, and enterohemorrhagic *Escherichia coli*. Foodborne Pathog. Dis..

[120] Matthysse A.G., Deora R., Mishra M., Torres A.G. (2008). The polysaccharides cellulose, poly-ß-1, 6-N-acetyl-D-glucosamine, and colanic acid are required for optimal binding of *E. coli* O157: H7 strains to alfalfa sprouts and K12 strains to plastic but not for binding to epithelial cells. Appl. Environ. Microbiol..

[121] Kang J., Li Q., Liu L., Jin W., Wang J., Sun Y. (2018). The specific effect of gallic acid on *Escherichia coli* biofilm formation by regulating *pgaABCD* genes expression. Appl. Microbiol. Biotechnol..

[122] Alibi S., Crespo D., Navas J. (2021). Plant-derivatives small molecules with antibacterial activity. Antibiotics.

[123] Jacob C., Melotto M. (2019). Human pathogen colonization of lettuce dependent upon plant genotype and defense response activation. Front. Plant Sci..

[124] Oblessuc P.R., Matiolli C.C., Melotto M. (2020). Novel molecular components involved in callose-mediated *Arabidopsis* defense against *Salmonella enterica* and *Escherichia coli* O157:H7. BMC Plant Biol..

[125] Ferelli A.M.C., Bolten S., Szczesny B., Micallef S.A. (2020). *Salmonella enterica* elicits and is restricted by nitric oxide and reactive oxygen species on tomato. Front. Microbiol..

[126] Schikora A., Garcia A.V., Hirt H. (2012). Plants as alternative hosts for Salmonella. Trends Plant Sci..

[127] Melotto M., Panchal S., Roy D. (2014). Plant innate immunity against human bacterial pathogens. Front. Microbiol..

[128] Jacob C., Velásquez A.C., Josh N.A., Settles M., He S.Y., Melotto M. (2021). Dual transcriptomic analysis reveals metabolic changes associated with differential persistence of human pathogenic bacteria in leaves of *Arabidopsis* and lettuce. G3 Genes Genomes Genet..

[129] Zarkani A.A., Schikora A. (2021). Mechanisms adopted by *Salmonella* to colonize plant hosts. Food Microbiol..

[130] Hunter P.J., Hand P., Pink D., Whipps J.M., Bending G.D. (2010). Both leaf properties and microbe-microbe interactions influence within-species variation in bacterial population diversity and structure in the lettuce (*Lactuca* species) phyllosphere. Appl. Environ. Microbiol..

[131] Pomposiello P.J., Bennik M.H., Demple B. (2001). Genome-wide transcriptional profiling of the *Escherichia coli* responses to superoxide stress and sodium salicylate. J. Bacteriol..

[132] Sulavik M.C., Gambino L.F., Miller P.F. (1995). The MarR repressor of the multiple antibiotic resistance (*mar*) operon in *Escherichia coli*: Prototypic member of a family of bacterial regulatory proteins involved in sensing phenolic compounds. Molec. Med..

[133] Fink R.C., Black E.P., Hou Z., Sugawara M., Sadowsky M.J., Diez-Gonzalez F. (2012). Transcriptional responses of *Escherichia coli* K-12 and O157: H7 associated with lettuce leaves. Appl. Environ. Microbiol..

[134] Van der Linden I., Cottyn B., Uyttendaele M., Vlaemynck G., Heyndrickx M., Maes M., Holden N. (2016). Microarray-based screening of differentially expressed genes of *E. coli* O157: H7 Sakai during preharvest survival on butterhead lettuce. Agriculture.

[135] Han S., Ferelli A.M.C., Lin S.-S., Micallef S.A. (2020). Stress response, amino acid biosynthesis and pathogenesis genes expressed in *Salmonella enterica* colonizing tomato shoot and root surfaces. Heliyon.

[136] Kyle J.L., Parker C.T., Goudeau D., Brandl M.T. (2010). Transcriptome analysis of *Escherichia coli* O157: H7 exposed to lysates of lettuce leaves. Appl. Environ. Microbiol..

[137] Das Q., Lepp D., Yin X., Ross K., McCallum J.L., Warriner K., Marcone M.F., Diarra M.S. (2019). Transcriptional profiling of *Salmonella enterica* serovar Enteritidis exposed to ethanolic extract of organic cranberry pomace. PLoS ONE.

[138] Goudeau D.M., Parker C.T., Zhou Y., Sela S., Kroupitski Y., Brandl M.T. (2013). The *Salmonella* transcriptome in lettuce and cilantro soft rot reveals a niche overlap with the animal host intestine. Appl. Environ. Microbiol..

[139] Kalily E., Hollander A., Korin B., Cymerman I., Yaron S. (2016). Mechanisms of resistance to linalool in *Salmonella* Senftenberg and their role in survival on basil. Environ. Microbiol..

[140] Le Bot J., Bénard C., Robin C., Bourgaud F., Adamowicz S. (2009). The ‘trade-off’ between synthesis of primary and secondary compounds in young tomato leaves is altered by nitrate nutrition: Experimental evidence and model consistency. J. Exp. Bot..

[141] Bénard C., Gautier H., Bourgaud F., Grasselly D., Navez B., Caris-Veyrat C., Weiss M., Génard M. (2009). Effects of low nitrogen supply on tomato (*Solanum lycopersicum*) fruit yield and quality with special emphasis on sugars, acids, ascorbate, carotenoids, and phenolic compounds. J. Agric. Food Chem..

[142] Marvasi M., George A.S., Giurcanu M., Hochmuth G.J., Noel J.T., Gause E., Teplitski M. (2014). Effects of nitrogen and potassium fertilization on the susceptibility of tomatoes to post-harvest proliferation of *Salmonella enterica*. Food Microbiol..

[143] Park M.-Y., Kang D.-H. (2021). Antibacterial activity of caffeic acid combined with ultraviolet-A light against *Escherichia coli* O157: H7, *Salmonella* Typhimurium and *Listeria monocytogenes*. Appl. Environ. Microbiol..

[144] Malheiro J.F., Maillard J.-Y., Borges F., Simões M. (2019). Biocide potentiation using cinnamic phytochemicals and derivatives. Molecules.

[145] Deng K., Wang S., Rui X., Zhang W., Tortorello M.L. (2011). Functional analysis of *ycfR* and *ycfQ* in *Escherichia coli* O157: H7 linked to outbreaks of illness associated with fresh produce. Appl. Environ. Microbiol..

[146] Crozier L., Hedley P.E., Morris J., Wagstaff C., Andrews S.C., Toth I., Jackson R.W., Holden N.J. (2016). Whole-transcriptome analysis of verocytotoxigenic *Escherichia coli* O157: H7 (Sakai) suggests plant-species-specific metabolic responses on exposure to spinach and lettuce extracts. Front. Microbiol..

[147] Toivonen P.M., Lu C., Bach S., Delaquis P. (2012). Modulation of wound-induced hydrogen peroxide and its influence on the fate of *Escherichia coli* O157: H7 in cut lettuce tissues. J. Food Prot..

[148] Khalil R.K., Frank J.F. (2010). Behavior of *Escherichia coli* O157: H7 on damaged leaves of spinach, lettuce, cilantro, and parsley stored at abusive temperatures. J. Food Prot..

[149] Gorski L., Flaherty D., Duhe J.M. (2008). Comparison of the stress response of *Listeria monocytogenes* strains with sprout colonization. J. Food Prot..

[150] Paudel S., Lin P.A., Foolad M.R., Ali J.G., Rajotte E.G., Felton G.W. (2019). Induced plant defenses against herbivory in cultivated and wild tomato. J. Chem. Ecol..

[151] Mauch-Mani B., Baccelli I., Luna E., Flors V. (2017). Defense priming: An adaptive part of induced resistance. Ann. Rev. Plant Biol..

[152] Chalupowicz L., Manulis-Sasson S., Barash I., Elad Y., Rav-David D., Brandl M.T. (2021). Effect of plant systemic resistance elicited by biological and chemical inducers on the colonization of the lettuce and basil leaf apoplast by *Salmonella enterica*. Appl. Environ. Microbiol..

[153] Hernandez-Reyes C., Schenk S.T., Neumann C., Kogel K.H., Schikora A. (2014). N-acyl-homoserine lactones-producing bacteria protect plants against plant and human pathogens. Microb. Biotechnol..

[154] Iniguez A.L., Dong Y., Carter H.D., Ahmer B.M., Stone J.M., Triplett E.W. (2005). Regulation of enteric endophytic bacterial colonization by plant defenses. Mol. Plant Microbe Interact..

[155] Marvasi M., Noel J.T., George A.S., Farias M.A., Jenkins K.T., Hochmuth G., Xu Y., Giovanonni J.J., Teplitski M. (2014). Ethylene signalling affects susceptibility of tomatoes to *Salmonella*. Microb. Biotechnol..

[156] Dang T.T., Shimatani Z., Kawano Y., Terada R., Shimamoto K. (2013). Gene editing a constitutively active OsRac1 by homologous recombination-based gene targeting induces immune responses in rice. Plant Cell Physiol..

[157] Wheatley M.S., Yinong Y. (2021). Versatile applications of the CRISPR/Cas toolkit in plant pathology and disease management. Phytopathology.

[158] Lu W., Deng F., Jia J., Chen X., Li J., Wen Q., Li T., Meng Y., Shan W. (2020). The *Arabidopsis thaliana* gene AtERF019 negatively regulates plant resistance to *Phytophthora parasitica* by suppressing PAMP-triggered immunity. Mol. Plant Pathol..

